# Splice site diversity and abundance of noncanonical introns in diplonemids (Diplonemea, Euglenozoa)

**DOI:** 10.1261/rna.080641.125

**Published:** 2025-12

**Authors:** Prasoon K. Thakur, Anzhelika Butenko, Filip Karásek, Michaela Svobodová, Drahomíra Faktorová, Hana Pavlisková, Vladimir Varga, Aleš Horák, Julius Lukeš, David Staněk

**Affiliations:** 1 Institute of Molecular Genetics, Czech Academy of Sciences, Prague, 142 20, Czech Republic; 2 Institute of Parasitology, Biology Centre, Czech Academy of Sciences, České Budějovice (Budweis), 370 05, Czech Republic; 3 Faculty of Science, University of South Bohemia, České Budějovice (Budweis), 370 05, Czech Republic; 4 Life Science Research Centre, Faculty of Science, University of Ostrava, Ostrava, 701 03, Czech Republic

**Keywords:** diplonemids, euglenozoans, noncanonical introns, snRNP, spliceosome

## Abstract

Noncoding introns are a unifying feature of protein-coding genes in virtually all extant eukaryotes, with most lineages following the canonical intron structure. However, euglenozoans, unicellular flagellates that include free-living euglenids, human pathogenic kinetoplastids, and highly diverse and abundant marine diplonemids, are a notable exception. Euglenozoan genomes range from extremely intron-poor kinetoplastids to euglenid genomes containing both canonical and noncanonical introns. Here, we present a comprehensive analysis of splice sites and spliceosomal components in six species of understudied diplonemids. All diplonemids examined contain a nearly complete set of spliceosomal snRNP components, indicating the presence of a functional U2-type spliceosome. However, the majority of introns in the hemistasiid diplonemids *Artemidia motanka* and *Namystynia karyoxenos* are noncanonical and lack conserved GT-AG terminal dinucleotides typical for U2-type introns. These noncanonical introns are capable of extensive base-pairing, which brings intron ends into close proximity. Thus, while the splicing apparatus is conserved in diplonemids, the splice sites are highly variable among individual species.

## INTRODUCTION

Many RNAs contain internal sequences that have to be removed before RNA is fully functional. These intervening segments called introns were found in most classes of RNA including rRNA, tRNA, and mRNA and can be divided into several categories. The first category consists of introns that are able to splice themselves out as ribozymes and are found in all three domains of life, as well as in eukaryotic organelles. They are further divided into two major groups I and II based on the nucleotides they use to initiate the splicing reaction (Nielsen and Johansen 2009; Pyle 2016). Next, there is a group of introns found in eukaryotic tRNAs. They are removed in several steps by a specialized machinery including a dedicated endonuclease and RNA ligase (Gerber et al. 2022). The last category represents introns found in most eukaryotic pre-mRNAs. These introns come in two flavors based on the sequences found at their ends. Major introns (also named U2-type introns) contain highly conserved GT and AG dinucleotides at the 5′ and 3′ ends, respectively. Minor introns (called U12-type) are more divergent and mostly contain AT and AC dinucleotides at their 5′ and 3′ termini (Patel and Steitz 2003; Wilkinson et al. 2020). Both major and minor introns are removed by large ribonucleoprotein complexes called spliceosomes. The spliceosome consisting of U1, U2, U4/U6, and U5 small nuclear ribonucleoproteins (snRNPs) is dedicated to the removal of U2-type introns, while U11, U12, U4atac/U6atac, and U5 snRNPs form the spliceosome catalyzing splicing of the U12-type introns (Kastner et al. 2019; Akinyi and Frilander 2021).

This classification was challenged by research on euglenids, a group of marine and freshwater flagellates, which revealed the existence of yet another group of noncanonical (also called nonconventional) introns in the protein-coding genes (Tessier et al. 1995; Breckenridge et al. 1999). The majority of introns in these protists belong to the canonical (GT-AG) type that can base pair with U1 snRNA, indicating their excision by the U2-type spliceosome (Breckenridge et al. 1999). However, due to the lack of base-pairing with U1 snRNA, the spliceosome does not seem to be involved in the excision of noncanonical introns usually flanked by short direct repeats allowing the formation of secondary structures that bring the 5′ and 3′ intron ends together (Muchhal and Schwartzbach 1992, 1994; Henze et al. 1995; Tessier et al. 1995; Milanowski et al. 2014, 2016; McWatters and Russell 2017). In addition, the noncanonical introns do not exhibit the structural features of group I and II self-splicing introns, and it is currently unclear how they are removed. The noncanonical introns are rather rare and usually found in unicellular eukaryotes. An exception is the chordate *Fritillaria borealis*, in which the majority of standard introns have been replaced by the noncanonical introns with highly divergent splice sites, but their removal likely occurs by the U2-type spliceosome (Henriet et al. 2019).

Protists of the phylum Euglenozoa are subdivided into the mostly parasitic Kinetoplastea and predominantly free-living Diplonemea and Euglenida (Flegontova et al. 2016; Obiol et al. 2020). As causative agents of serious human diseases, kinetoplastids belong to the best studied protists, while their sister clade of diplonemids remained neglected. Yet, diplonemids recently emerged as one of the most abundant, diverse, and speciose groups of marine eukaryotes, thus of major significance for the oceanic ecosystem (Flegontova et al. 2016, 2020; Schoenle et al. 2021). Several diplonemid species have been introduced into culture, allowing the generation of numerous transcriptomes, complemented by a high-quality genome assembly of the model species *Paradiplonema papillatum* (Valach et al. 2023b). Moreover, this diplonemid became genetically manipulatable (Faktorová et al. 2020), allowing in-depth studies, such as the characterization of its mitochondrial ribosome (Valach et al. 2023a), unusual kinetochores (Benz et al. 2024), and trafficking machinery (Faktorová et al. 2023; Záhonová et al. 2025). Despite these advances, our understanding of diplonemid biology remains rather limited.

The first attempt to characterize the architecture of nuclear genes of diplonemids was based on fragmentary genomic sequences obtained by single-cell sequencing of 10 unclassified marine diplonemids (Gawryluk et al. 2016). The analysis unveiled a considerable number of putative introns, which came as a surprise, since the sister clade of diplonemids, the well-studied kinetoplastids, are known to contain only three protein-coding genes with introns (Schneider et al. 1994; Mair et al. 2000; Kostygov et al. 2024). Moreover, diplonemid introns were predicted, in the absence of transcriptomic data, to be noncanonical, not adhering to the typical GT-AG intron boundaries (Gawryluk et al. 2016). However, recent systematic and thorough analysis of *P. papillatum* revealed a predominant presence of canonical GT-AG introns with a minority of the noncanonical introns featuring the GC-AG splice-site combination (Valach et al. 2023b).

In this study, we analyzed introns from six species representing a substantial fraction of known diplonemid diversity, namely *Artemidia motanka* and *Namystynia karyoxenos* (family Hemistasiidae)*,* and *Sulcionema specki, Lacrimia lanifica, Diplonema japonicum,* and *Rhynchopus humris* (family Diplonemidae). Mapping RNA-seq reads on the draft genome assembly allowed the identification and basic characterization of their introns. In addition, we monitored the presence of all major components of the spliceosome. Our data show a considerable heterogeneity of gene architecture in diplonemids and provide an insight into the unusual intron evolution in these ecologically and evolutionary important yet understudied protists.

## RESULTS

### Canonical and noncanonical introns in diplonemid genomes

Draft genome assemblies were generated for *A. motanka*, *N. karyoxenos*, *S. specki*, *L. lanifica*, *D. japonicum*, and *R. humris*. The genome assembly sizes ranged between 35 and 118 Mb for *S. specki* and *N. karyoxenos*, respectively, and N_50_ values varied between 1160 bp for *N. karyoxenos* and 11,390 bp for *L. lanifica* (Supplemental Table S1). Genome assembly statistics were assessed using BUSCO (Seppey et al. 2019) and KAT methods (Supplemental Table S2; Mapleson et al. 2017). Next, we performed intron prediction aided by mapping RNA-seq data to the genome assemblies (see Supplemental Fig. S1; Materials and Methods for details). To ensure the acquisition of high-confidence intron models, we excluded introns supported by fewer than 15 split reads. This filtering process yielded 98,221 introns from *A. motanka*, 21,076 from *N. karyoxenos*, 60,346 from *S. specki*, 12,835 from *L. lanifica*, 10,131 from *D. japonicum*, and 13,462 from *R. humris*.

**FIGURE 1.**
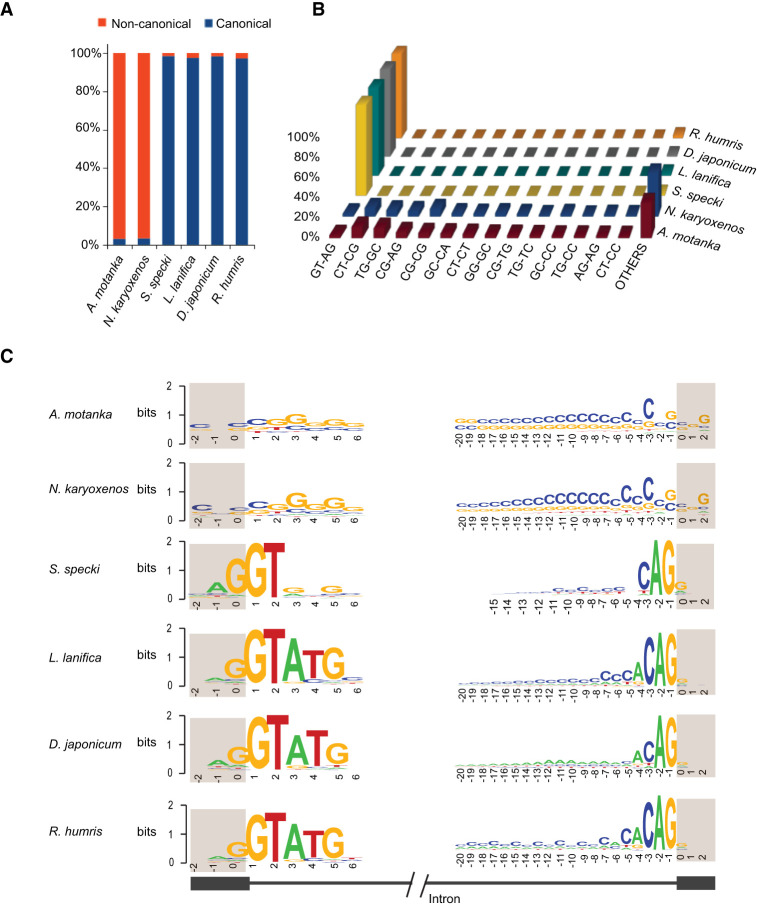
Splice-site sequences in diplonemids. (*A*) Stacked bar plot shows the percentage of canonical and noncanonical introns. (*B*) Distribution of splice site sequences. First two nucleotides from 5′ end and last two nucleotides from 3′ end of introns are shown. (*C*) Sequence logos of 5′ and 3′ splice sites.

Next, we aimed to identify and characterize common intronic motifs across these six diplonemid species. We categorized the introns based on their splice site sequences and those possessing GT and AG dinucleotides at their 5′ and 3′ splice site, respectively, classified as canonical introns. Conversely, introns with the splice sites deviating from the canonical “GT-AG” motif were classified as noncanonical introns. We observed an overwhelming dominance of canonical introns in *S. specki* (98.44%), *L. lanifica* (97.45%), *D. japonicum* (98.33%), and *R. humris* (97.10%), reaffirming the prevailing trend observed in the wide majority of eukaryotes. However, the picture was very different in two Hemistasiidae species, *A. motanka* and *N. karyoxenos*, as these diplonemids turned out to be extremely rich in the noncanonical splice sites, which constituted 96.93% and 96.64% of all splice sites, respectively (Fig. 1A). Further analysis of these noncanonical splice site categories revealed a diverse landscape of dinucleotides at the intron ends. Most common combinations were CT-CG, TG-GC, CG-AG, and GC-CA, each amounting to <12% of all the identified intron ends (Fig. 1B; Supplemental Fig. S2A,B), which indicates a lack of splice site conservation.

We assessed intron length distributions across six diplonemid species (Supplemental Fig. S3A). The median intron length in noncanonical species was 91 and 37 nt in *N. karyoxenos* and *A. motanka*, respectively. When separating canonical and noncanonical introns, *N*. *karyoxenos* displayed longer canonical introns (105 nt) compared to noncanonical ones (91 nt). In *A. motanka*, canonical introns were 10% longer (41 nt) than noncanonical (37 nt) introns. The diplonemid species with canonical introns showed similar intron length distribution: *L. lanifica* had the longest introns with a median length of 107 nt, while *D. japonicum* and *S. specki* had shorter introns with medians of 41 and 42 nt, respectively. *R. humris* exhibited a moderate intron length, with a median of 74 nt. However, as the analyzed genomes are not complete and large part, namely repetitive sequences are missing, these intron length distributions may not fully represent the complete genomic architecture of each species, and especially long introns could have been missed.

In the following step, we analyzed intronic splicing signals—including the 5′ splice site (5′ss), branch point, and polypyrimidine tract/3′ splice site (PPT/3′ss)—using all introns regardless of their canonical or noncanonical classification (Fig. 1C). The primary aim of this analysis was to uncover the consensus sequence and the extent of conservation in the proximity of the splice sites. The 5′ss included 3 bases at the 3′ end of upstream exon and 6 bases at the 5′ end of intron, while the 3′ss was defined as 20 bases in the intron and 3 bases in the downstream exon. In *L. lanifica*, *D. japonicum*, and *R. humris*, the most frequent nucleotides were (G)/GTATG at the 5′ss, while only a shorter G/GT motif is present in *S. specki* (Fig. 1C). The 5′ss region was strikingly different in *A. motanka* and *N. karyoxenos* and displayed a strong preference for C and G nucleotides (Fig. 1C).

To identify the branch point signature, using all introns combined, we searched for the YTRAY motif within intronic region 5–50 bp upstream of the 3′ss (see seqlogo in Supplemental Fig. S4A). The proportion of introns containing this motif was rather low, which might reflect the high variability of the sequence surrounding the branch point. The presence of the branch point motif varied among species, with 2.4%, 6.8%, 8.3%, 15.8%, 13.9%, and 13.1% detected in *A. motanka*, *N. karyoxenos*, *S. specki*, *L. lanifica*, *D. japonicum*, and *R. humris*, respectively. The analysis of PPT sequence revealed a preference for C in the pyrimidine tract of each species, except for *D. japonicum*, where, unusually, A was the most abundant nucleotide. Weak presence of A in the PPT-3′ss was also found in *L. lanifica* and *R. humris. S. specki* exhibited the shortest PPT primarily comprised of pyrimidine (C and T) residues.

Finally, we analyzed the 3′ end of introns. Four Diplonemidae species have a canonical CAG sequence at this site, while there is no strong bias for a particular sequence at the 3′ ends of introns of the two examined representatives of Hemistasiidae. To search for specific motifs that can navigate the splicing machinery, we performed de novo motif discovery (MEME) on the complete set of introns in each species (Bailey and Elkan 1994). The analysis revealed CA- or CAC-rich motifs in *A. motanka*, *N. karyoxenos*, *R. humris*, and *L. lanifica*. In *S. specki*, two distinct motifs were identified—one enriched in CA repeats and another with G-rich content. These motifs varied in length and composition, suggesting species-specific conservation. Full motif logos are shown in Supplemental Figure S4B. Combined, these data suggest that intronic splicing signals in diplonemids are diverse and species-specific, with a particularly distinct noncanonical intron-defining patterns in *A. motanka* and *N. karyoxenos*.

### Validation of intron predictions through RT-PCR analysis

To test the accuracy of our intron annotation, we used two alternative approaches. First, we reanalyzed recently published transcriptomic and genomic data of *P. papillatum* (Valach et al. 2023b). We applied our pipeline to raw transcriptomic and genomic reads from *P. papillatum* to identify introns de novo. Then, we created splice site sequence logos from the intron annotations obtained from our pipeline and compared them with logos derived from the previously published annotation (Valach et al. 2023b). This comparison revealed a strong similarity between the annotations, indicating that our bioinformatics analyses are able to adequately identify introns (Supplemental Fig. S3B).

**FIGURE 2.**
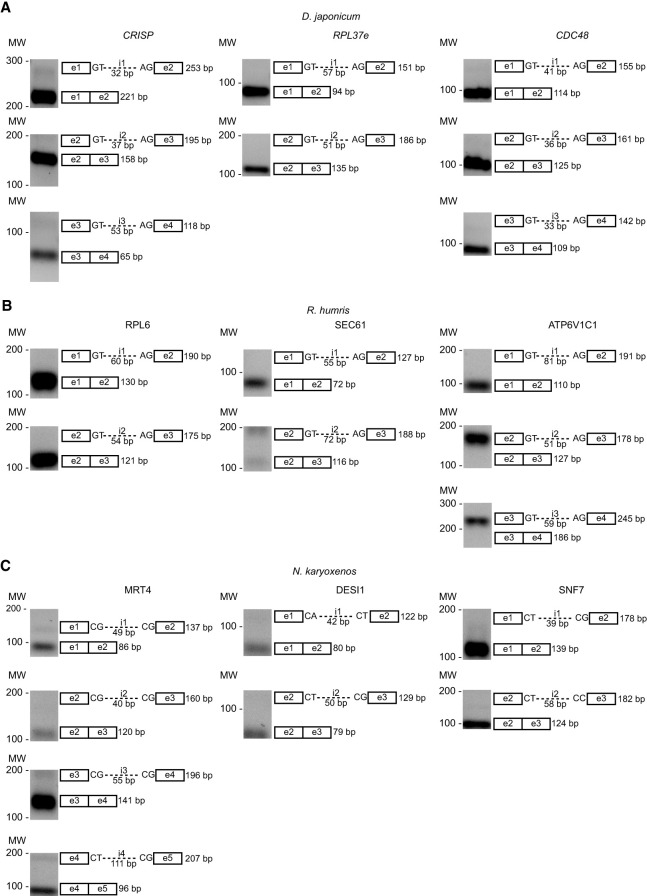
Detection of selected mRNAs in three diplonemid species. (*A*) *D. japonicum* (*CRISP*, *RPL37e*, *CDC48*), (*B*) *R. humris* (*RPL6*, *Sec61*, *ATP6V1C1*), and (*C*) *N. karyoxenos* (*MRT4*, *DESI1*, *SNF7*). Gel images are presented with corresponding exon (e)–intron (i) structures indicating splice site boundaries and the expected size of spliced and unspliced products. Exons are depicted as boxes and introns as lines.

Second, we isolated total RNA from *D. japonicum*, *R. humris*, and *N. karyoxenos* and subjected it to RT-PCR in order to experimentally validate some of the predicted canonical and noncanonical introns. Analysis of eight canonical introns from three genes of *D. japonicum*, cysteine-rich secretory protein (*CRISP*), ribosomal protein L37e (*RPL37e*), and cell division control protein 48 (*CDC48*), revealed products that corresponded to the predicted size of mRNA after removal of the introns (Fig. 2A). The same approach demonstrated efficient removal of four out of seven canonical introns from the following *R. humris* genes: ribosomal protein L6 (*RPL6*), transport protein Sec61 (*SEC61*), and V-type proton ATPase subunit C1 (*ATP6V1C1*) (Fig. 2B). Finally, RT-PCR analysis of eight noncanonical introns predicted in three genes in *N. karyoxenos* Turnover 4 homolog (*MRT4*), desumoylating isopeptidase 1 (*DESI1*), and vacuolar protein sorting-associated protein 32 (*SNF7*) also confirmed the presence of spliced products (Fig. 2C). Combined, these results are consistent with our intron annotations in these diplonemid species.

### Exploring splicing in diplonemids using reporter minigene assays

Due to the inaccessibility of diplonemids to genetic manipulation, we investigated intron splicing using the related and experimentally tractable kinetoplastid *Trypanosoma brucei*. We expressed three *N. karyoxenos* genes (*MRT4*, *POC1*, and *GK*) containing both canonical and noncanonical introns, and one canonical intron-containing gene (*MRT4*) from *P. papillatum* in *T. brucei* under inducible promoter. We monitored the presence of spliced products before and 24 h after induction. Spliced mRNA was detected only for the canonical GK intron 1, which was confirmed by sequencing (Supplemental Fig. S5B,C). No spliced products were observed for the noncanonical introns, and the unexpected band from GK intron 2 did not match the predicted splice product. We also attempted to express *N. karyoxenos* reporter genes in *P. papillatum* but successfully generated a stable line only for POC1. No spliced transcripts were detected (Supplemental Fig. S5D). Together, these findings indicate that *T. brucei*’s splicing machinery processes diplonemid introns inefficiently and that *P. papillatum* may have different intron recognition requirements.

### Characterization of the spliceosome components in diplonemids

**FIGURE 3.**
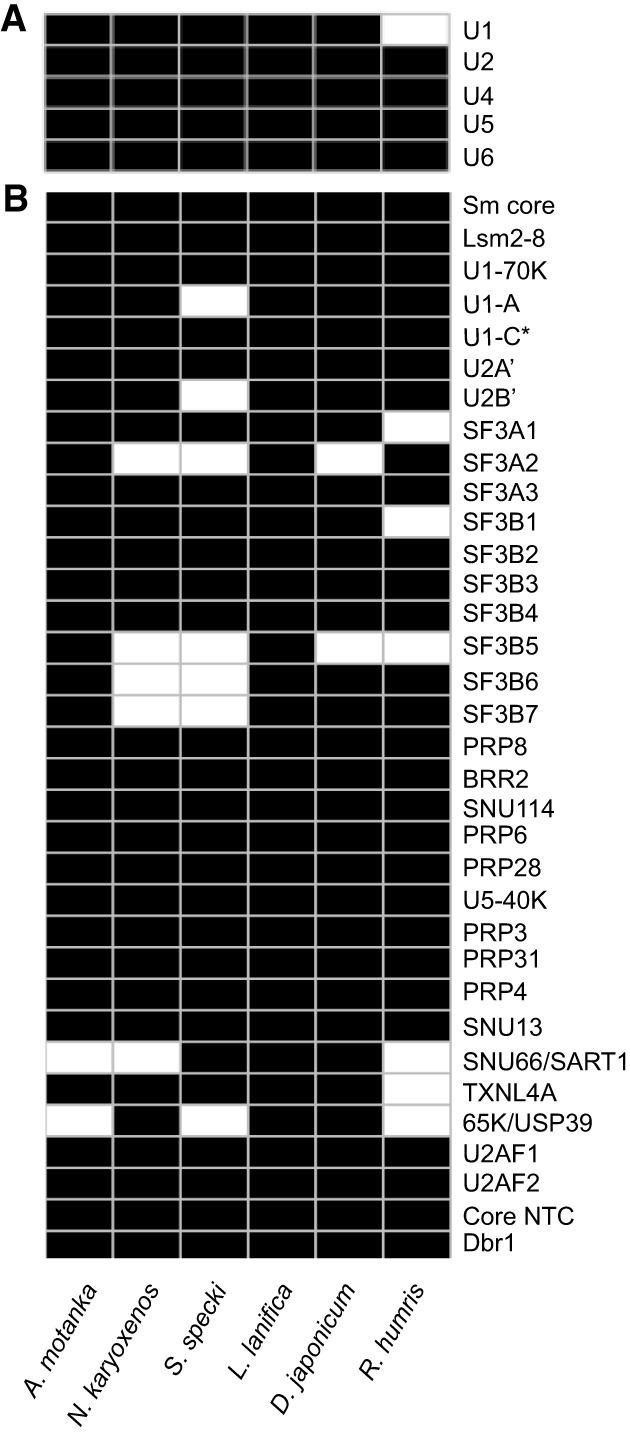
Identification of spliceosomal components in diplonemids. (*A*) Identified spliceosomal snRNAs. (*B*) Identified orthologs of spliceosomal proteins, including the Sm core proteins (SmB/B′, D1, D2, D3, E, F, and G), and core NTC components (PRP19, Celf1/CDC5L, Syf1/NTC90, Syf2/NTC31, Isy/NTC30, PRP1/PRPG1, and Spf27/NTC25). Black boxes indicate the presence of the snRNA/protein, and asterisks (*) denote partially identified proteins. Notably, Isy/NTC30 was not detected in *N. karyoxenos*, Syf1/NTC90 in *A. motanka*, and Syf2/NTC31and SmE in *R. humris*.

**FIGURE 4.**
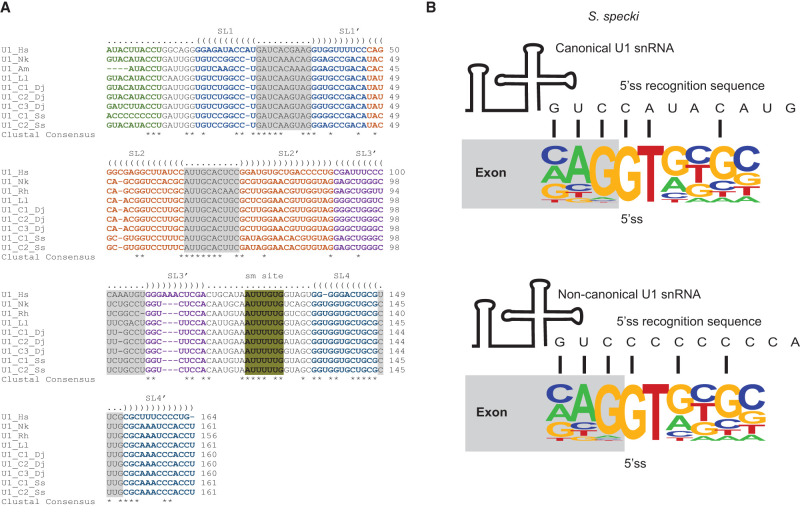
U1 snRNA variants in diplonemids. (*A*) Multiple sequence alignment of U1 snRNA from various diplonemid species and *Homo sapiens* used as a reference. Each sequence is labeled with the species abbreviation followed by “C1” or “C2” or “C3” to indicate variant number. Species abbreviations: *A. motanka* (Am), *N. karyoxenos* (Nk), *S. specki* (Ss), *L. lanifica* (Ll), *D. japonicum* (Dj), *R. humris* (Rh), and *H*. *sapiens* (Hs). Consensus sequences are indicated below each alignment. Sm-sites are indicated in olive color. (*B*) Schematic representation of potential base-pairing between U1 snRNA and 5′ss sequence in *S. specki*. The *top* panel displays the canonical U1 snRNA, and the *bottom* panel depicts the noncanonical U1 snRNA along with the sequence logo of the 5′ss.

The prevalence of noncanonical introns in Hemistasiidae leads us to investigate whether the splicing machinery differs among diplonemids. We identified all snRNAs at the genomic level in the assayed diplonemid genomes except in *R. humris* in which we could not find U1 snRNA (Fig. 3A; Supplemental Data S1). Based on homology, all identified snRNA genes belong to the U2 type, rather than the U12 type (Supplemental Figs. S6–S11). Interestingly, *A. motanka* and *N. karyoxenos,* which contain a high proportion of the noncanonical introns (Fig. 1), encode well-conserved U1 snRNA genes that are complementary to canonical but not to noncanonical splice sites. However, we cannot rule out the possibility that *A. motanka* and *N. karyoxenos* also contain noncanonical U1 snRNAs, which could not be identified due to their extreme divergence or the incompleteness of the genome assemblies. We found three and two copies of U1 snRNA in the *D. japonicum* and *S. specki* genomes, respectively (Fig. 4A). A more detailed inspection of the genomic U1 snRNA sequences revealed that U1 snRNAs in *S. specki* differ in the 5′ end sequence that base pairs with the 5′ss. One U1 snRNA gene contains a conserved sequence at the 5′ end, which is replaced by a stretch of Cs in the other U1 snRNA gene (Fig. 4). However, an examination of the potential base-pairing between the 5′ss of both the canonical and alternative U1 snRNAs showed comparable complementarity with the canonical 5′ss sequence logo identified in *S. specki* (Fig. 4B).

Search for the protein components of spliceosomal snRNPs, which are the basic building blocks of the spliceosome, led to the identification of most human orthologs found in mammalian spliceosomal snRNPs (Fig. 3B; Will and Lührmann 2011). Notably, we found that only ∼55 amino acids at the N terminus of the SNRPC (U1-C) protein that interact with U1 snRNA are conserved. To provide a more complete view of the spliceosome components, we extended our search to include additional conserved and functionally critical spliceosomal proteins such as U2AF1/2, Dbr1, and components of the NineTeen complex. Interestingly, U2AF1/2 were found also in *D. japonicum*, which contains the atypical poly(A) tracts in PPT. Together, these findings reveal that diplonemids possess a conserved core of spliceosomal RNAs and proteins. Despite some unique sequence features and intron structures, the presence of key snRNPs, U2AF, and additional spliceosomal components supports the existence of a functional spliceosome capable of recognizing and excising canonical introns across diplonemid species.

### High base-pairing of noncanonical splice sites

**FIGURE 5.**
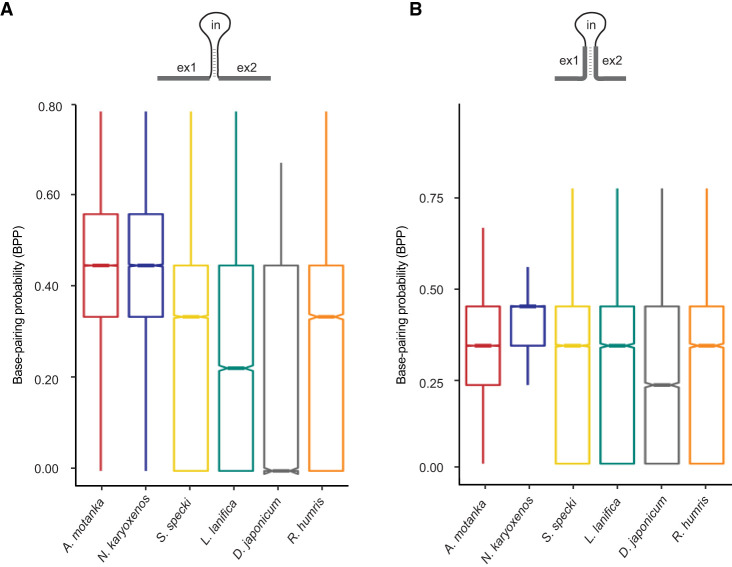
Base-pairing probability (BPP) between 5′ and 3′ splice sites in various diplonemid species. (*A*) BPP was calculated for intronic sequences: 9 nt at the 5′ end of the intron and 9 nt at the 3′ end of the same intron. (*B*) BPP was calculated for exonic sequences: 9 nt at the 3′ end of the upstream exon and 9 nt at the 5′ end of the downstream exon. In both cases, BPP is defined as the number of paired nucleotides between the two 9 nt regions, divided by 18. Box plots show the distribution of BPP values across species. The boxes represent the interquartile range, the horizontal line indicates the median, and the notches denote the 95% confidence interval around the median.

An overrepresentation of G and C nucleotides at both splice sites in *A. motanka* and *N. karyoxenos* (Fig. 1C) prompted us to investigate the base-pairing probability between the intron ends in all studied diplonemids. The results show a higher propensity for base-pairing between nine terminal nucleotides of introns in *A. motanka* and *N. karyoxenos* compared to other diplonemids with canonical splice sites (Fig. 5A). To assess whether this trend also extends to exonic regions, we examined the 9 nt upstream of the 5′ splice site and the 9 nt downstream from the 3′ss. The exonic sequences surrounding noncanonical introns also showed a tendency for higher base-pairing, but the difference between canonical and noncanonical species was smaller than in case of introns. (Fig. 5B). To investigate the base-pairing probability between the 5′ and 3′ ends of introns of *A. motanka* and *N. karyoxenos* in more detail, secondary structure predictions have been performed for a subset of introns exhibiting high base-pairing probability. Indeed, in these two diplonemids, strong base-pairing between both ends of introns has been revealed (Supplemental Figs. S12, S13). This finding suggests that the 5′ and 3′ end base-pairings facilitate secondary structure folding of introns, which could be crucial for the splicing of noncanonical introns in these marine protists.

### Splice site analysis by comparative genomics

**FIGURE 6.**
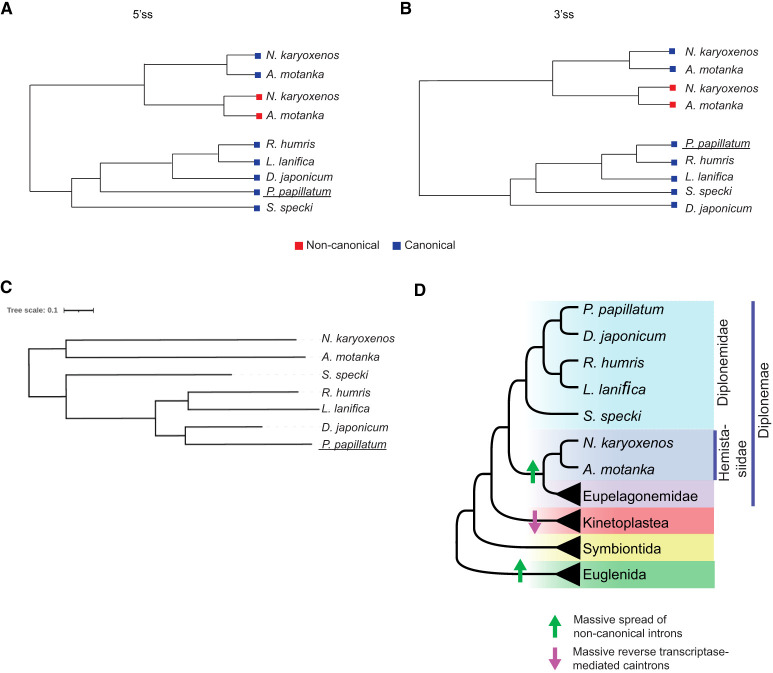
Clustering of diplonemids based on their splice site sequences. Phylogenetic clustering of 5′ (*A*) and 3′ splice site (*B*) motifs of canonical and noncanonical introns from *A. motanka* and *N. karyoxenos* with other diplonemid species. Position weight matrices were used to construct phylogenetic trees for the splice sites. (*C*) Phylogenomic tree predicted from transcriptomic data of seven diplonemid species (*A. motanka*, *N. karyoxenos*, *S. specki*, *L. lanifica*, *D. japonicum*, *R. humris*, *P. papillatum*). Scale bar represents 0.1 substitutions per site. (*D*) Cladogram of Euglenozoa depicting major events in intron evolution. Cladogram is based on the most recent phylogenomic studies (Kostygov et al. 2021). Massive spread of noncanonical introns likely occurred in the common ancestor of eupelagonemids (Gawryluk et al. 2016) and hemistasiids. Massive intron loss in the kinetoplastid common ancestor was likely mediated by reverse transcriptases (Kostygov et al. 2024).

To trace the potential origin of the noncanonical introns using a phylogenetic approach, we compared the evolutionary trends of canonical and noncanonical splice sites of *A. motanka* and *N. karyoxenos* with other marine diplonemids including the recently sequenced *P. papillatum* (Valach et al. 2023b). Moreover, to avoid masking signals from other splice site positions, conserved dinucleotides at the intron ends have been removed from the analysis. Position weight matrices (PWMs) for both the 5′ss and 3′ss were used to compute a distance matrix between species. Resulting phylogenetic trees generated for both canonical and noncanonical splice sites revealed distinct clustering of the 5′ss and 3′ss, reflecting evolutionary relationships among these splice sites across species. Such a distinct clustering of canonical and noncanonical introns indicates that the noncanonical introns appeared in Hemistasiidae after the split of this family and Diplonemidae. Consistently, a comparative analysis of intron positions among *A. motanka*, *N. karyoxenos*, and *P. papillatum* revealed no evidence of conserved intron locations or of canonical introns being replaced by noncanonical introns. Canonical and noncanonical splice sites of *A. motanka* and *N. karyoxenos* cluster together, which suggest their common origin or less likely their convergent evolution (Fig. 6A,B). Although *A. motanka* and *N. karyoxenos* are sister clades with long branches, mutual clustering of their canonical and noncanonical splice sites indicates coevolution of these sequence elements. Finally, we compared the branching order of diplonemids using our transcriptomic data (Fig. 6C) with sorting based primarily on 18S rRNA sequences (Fig. 6D; Tashyreva et al. 2022). These data are consistent with and confirm the evolutionary position of *S. specki* between Hemistasiidae and Diplonemidae species.

## DISCUSSION

Only recently have the neglected diplonemid flagellates not only proven to be ecologically highly relevant, but also turned out to be very interesting organisms from an evolutionary point of view. Few in-depth studies focusing on their molecular aspects, such as RNA editing, mitochondrial genome structure, kinetochores, mitochondrial ribosomes, and the trafficking machinery (Burger and Valach 2018; Lukeš et al. 2018; Kaur et al. 2020; Valach et al. 2023a; Záhonová et al. 2025), showed that diplonemids belong to the representatives of “extreme” biology. While some of these systems may trace their origins back to last eukaryotic common ancestor (LECA) (e.g., the organization of the trafficking complexes [Záhonová et al. 2025]), their present-day forms in diplonemids are highly derived, suggesting a complex interplay of ancestral retention and lineage-specific innovation.

It seemed therefore likely that their genetic structure was also noncanonical. While the single complete genome and a handful of partial genomes and transcriptomes were available for this diverse group of protists, their analysis unearthed rare RNA processing events such as *trans*-splicing, extensive RNA editing, and unconventional intron boundaries, including introns that lacked the consensus sequences at their 5′ and 3′ ends. While the majority of introns in their nuclear-encoded genes have the canonical GT-AG boundaries, substantial fraction of them are noncanonical, reminiscent of introns described in the related euglenids (Milanowski et al. 2014; Valach et al. 2023b). Moreover, preliminary analyses of single-cell sequencing data of the most diverse and abundant diplonemid lineage—the eupelagonemids, sampled in the northern Pacific, indicated a prevalence of the noncanonical introns (Gawryluk et al. 2016).

Here, we analyzed the draft genome and transcriptome assemblies of six diplonemid species to determine the identity of their introns. While canonical introns clearly predominated in four members of Diplonemidae family, most introns found in two members of the family Hemistasiidae fall into the noncanonical category. Since Hemistasiidae are recognized as a sister group of eupelagonemids mentioned above, this prevalence of noncanonical introns seems to be a phylogenetic feature defining these two diplonemid clades. To find out whether the same differences also occur in their splicing machinery, we mapped the splicing factors in all examined species. We found no significant differences in their major snRNAs and snRNP-specific proteins indicating that all examined species have a complete and functional splicing apparatus. In addition to a classical U1 snRNA, we identified alternative U1 snRNA in *S. specki*. The alternative U1 snRNA contains an unusual stretch of C at the 5′ end and has the capacity to base pair with G-rich 5′ss sequences. This may indicate an adaptation of the splicing machinery to recognize degenerated 5′ss.

At the same time, we did not identify any alternative U1 snRNA with a potential to interact with the G-rich 5′ss of *N. karyoxenos* and *A. motanka*. This indicates that in these two species, the spliceosome, or at least the U1 snRNP, is not involved in the splicing of noncanonical introns, leaving the question about the mechanism of their removal open. However, the answer may lie in their secondary structure, as the noncanonical introns of *N. karyoxenos* and *A. motanka* have a significantly higher potential for the formation of secondary structures than introns in the other diplonemids. A closer look at representative noncanonical introns revealed strong base-pairing between their 5′ and 3′ ends (Supplemental Figs. S11, S12), which is in good agreement with previous reports that predicted a high probability of their secondary structure formation (Milanowski et al. 2014, 2016; Gawryluk et al. 2016). Here, we show that this feature is indeed a general property of the noncanonical introns with the 5′ and 3′ ends interacting with each other.

Recent studies of the model euglenid *Euglena gracilis* showed that the noncanonical introns are spliced with different kinetics than their canonical counterparts. In addition, after their removal, the noncanonical introns form circular RNA, which indicates spliceosomal-independent pathway of their excision (Guminska et al. 2021). How are then the noncanonical introns removed? We propose several potential mechanisms. One possibility is that proteins from the RNase III family, which efficiently recognize and cleave RNA duplexes, first excise the introns. The resulting exon termini could then be joined by an RNA ligase to generate a continuous mRNA, while the free intron ends could be ligated into circular RNA molecules.

Alternatively, noncanonical introns might be processed by a specialized splicing machinery that evolved from the pathway removing introns from highly structured tRNAs. In support of this idea, excised tRNA introns in *Drosophila melanogaster* form circular RNAs (Lu et al. 2015), as also observed for noncanonical introns in *E. gracilis* (Guminska et al. 2021). However, it should be noted that we did not find any structural similarities between noncanonical introns and tRNAs.

A third possibility is that these introns are removed by the classical spliceosome, which is well conserved in all six diplonemid species examined. In chordates, the spliceosome of *F. borealis* has been suggested to adapt for the removal of noncanonical introns (Henriet et al. 2019). Our study indicates that the splicing machinery in species with predominantly canonical introns is also flexible Notably, while PPT typically contains the pyrimidine bases U or C, we observed significant conservation of the purine base A within the PPT of *D. japonicum* (Fig. 1). Taken together, these findings are consistent with a highly adaptable diplonemid spliceosome that may also have evolved the capacity to splice noncanonical introns.

Genomes of all extant organisms are under constant pressure from transposable elements inserting into essential genes and disrupting their expression (Kazazian 2004; Feschotte and Pritham 2007). Hence, these genetic elements, such as the MITE-like transposons, may serve as the source of noncanonical introns (Feschotte and Pritham 2007). Moreover, since the 5′ and 3′ intron ends of *N. karyoxenos* and *A. motanka* contain inverted G- and C-rich repeats, it is plausible to assume that abundant noncanonical introns originated from the insertion of MITE-like transposons (Milanowski et al. 2014). The ecological success and evolutionary expansion, combined with gene-rich genomes coding for a very broad metabolic repertoire and the LECA-type features, testify of exceptional adaptability of these marine flagellates. They might have as well successfully adapted to the invasion of intervening sequences either by altering their splicing machinery, as seems to be the case in the multicellular *F. borealis* (Henriet et al. 2019), or by using different, so far uncharacterized strategies, as likely occurred in *E. gracilis* (Guminska et al. 2021), and *A. motanka* and *N. karyoxenos* (this study). At any rate, it is intriguing that the nuclear protein-coding genes of diplonemids are literally packed with introns, while their sister clade of kinetoplastids contains almost no introns in the nuclear genome (Kostygov et al. 2024). In addition, out of a narrow set of available species, one diplonemid carries almost exclusively canonical introns, while the ratio is reverted in favor of the noncanonical introns in another species. Thus, the complexity of intron evolution and its outcomes in Euglenozoa, particularly in diplonemids, matches or even surpasses that observed in multicellular organisms (Henriet et al. 2019). This attests to the remarkable genome evolvability and adaptability of these protists.

## MATERIALS AND METHODS

### Cell cultivation

The axenic cultures of *S. specki*, *L. lanifica*, *R. humris*, *D. japonicum*, *A. motanka*, and *N. karyoxenos* were grown in an artificial medium composed of 3.6% sea salts (Sigma-Aldrich), 1% (v/v) heat-inactivated horse serum (Sigma-Aldrich), and 0.025 g/L LB broth powder (Amresco), as described previously (Tashyreva et al. 2018; Prokopchuk et al. 2019). *P. papillatum* (ATCC 50162) was cultivated axenically following a protocol described previously (Kaur et al. 2018).

### RNA and DNA isolation for library preparation and sequencing

For each diplonemid species, DNA and RNA for genomic and RNA-seq sequencing were isolated using the standard phenol-chloroform protocol and the NucleoSpin RNA isolation kit (Macherey-Nagel), respectively. Paired-end 250 bp Illumina MiSeq reads were obtained using the standard TruSeq protocol. For transcriptome sequencing, paired-end 100 bp reads were sequenced on Illumina HiSeq 4000 platform using the standard TruSeq protocol. The sequencing was carried out by Genome Quebec.

### De novo genome and transcriptome assembly and annotation

The overlapping paired-end DNA reads were merged using bbmerge (BBtools v.38.90) (Bushnell et al. 2017) with default settings. The combination of merged and not merged reads was used for the genome assembly using SPAdes v.3.15.5 (Prjibelski et al. 2020) under default parameters. Further, genome assembly completeness was evaluated using two complementary approaches: Benchmarking Universal Single-Copy Orthologs (BUSCO) and K-mer Analysis Toolkit (KAT). BUSCO v5.7.1 (Waterhouse et al. 2019) was run in genome mode *alveolata_odb10* lineage data sets to assess the presence of conserved single-copy orthologs. While the *euglenozoa_odb10* data set includes 130 BUSCOs and is tailored to Euglenozoa, it is heavily biased toward trypanosomatids. Therefore, the *alveolata_odb10* data set (171 BUSCOs) was also used to provide a broader perspective on gene content completeness. In parallel, k-mer-based completeness was evaluated using KAT v2.4.2 (Mapleson et al. 2017) with a k-mer size of 21. KAT comp was used to compare raw genomic reads to the assembled genomes, allowing us to estimate the fraction of unique k-mers from the reads that are present in the assembly. These results provide an assembly-specific estimate of genome completeness, independent of gene prediction. The results of both analyses are summarized in Supplemental Table S2. Similarly, for paired-end RNA-seq reads, bbmerge was used, followed by the assembly using rnaSPAdes (Bushmanova et al. 2019). The transcriptome assembly was subsequently aligned to the assembled genome using GMAP (version 2021-02-12) (Wu and Watanabe 2005), using the parameter ‐‐canonical-mode=0 to generate preliminary gene models for each species. Additionally, paired-end RNA-seq reads were aligned to their respective assembled genomes. STAR aligner v.2.7.8a (Dobin et al. 2013) was used for this purpose, with and without reference annotation, using a two-pass mode. Other parameters were as follows: alignIntronMin=12, outFilterMismatchNoverReadLmax=0.02, scoreGapNoncan=-4, scoreGapATAC=-4, and alignIntronMax=10,000. The output of STAR alignment, both with and without gmap reference annotation, was used for transcript reconstruction via StringTie v.2.1.4 with -rf flag and guided by reference annotation.

Subsequently, both GTF files derived from StringTie, representing transcript annotation, were used as an input for the gffcompare program v.0.12.2 with the strict-match parameter to generate consensus annotations. Transcripts exhibiting “matching” intron chains with the class code “=” were selected for each species, and the introns lacking strand information were excluded from the analysis. Overlapping introns were filtered during the analysis.

In addition, we reannotated *A. montanka* and *N. karyoxenos* using stringent criteria to refine the alignment process, considering the high abundance of noncanonical introns in both species. The alignment was performed with the following STAR parameters: alignIntronMin 20, alignIntronMax 10000, outFilterMismatchNoverReadLmax 0.02, outFilterMismatchNmax 3, outFilterMultimapNmax 1, scoreGapNoncan -6, scoreGapATAC -6, chimSegmentMin 20, chimJunctionOverhangMin 20, outSJfilterOverhangMin 6 6 6 6, outSJfilterDistToOtherSJmin 5 5 10 10. The alignment was performed in “End-to-End” mode (alignEndsType EndToEnd). Similar to the previous run, the output of the STAR alignment, both with and without gmap reference annotation, was used for transcript reconstruction with StringTie v2.1.4. The following parameters were applied: m 200, f 0.2, j 8, g 50, c 3, A 5, and rf. Both GTF files generated by StringTie, which represent the transcript annotations, were used as input for the gffcompare program with the strict-match setting to create consensus annotations. For each species, transcripts with “matching” intron chains (class code “=”) were selected, and introns without strand information were excluded from the analysis. Overlapping introns were filtered during the analysis. Both annotations produced similar results; however, the second annotation run was selected for downstream analysis of *A. montanka* and *N. karyoxenos*.

To further refine the results, split reads in the BAM files representing exon–exon boundaries were counted using BEDTools v.2.30.0 intersect function, and the boundaries supported by fewer than 15 split reads were discarded. The agat_sp_add_introns tools v.0.7.0 (https://agat.readthedocs.io/en/latest/tools/agat_sp_add_introns.html) was used to augment intron features to the consensus GTF file. Subsequently, 5′ splice sites (3 bp upstream and 6 bp downstream) and 3′ splice sites (20 bp upstream and 3 bp downstream) were extracted from the intron coordinates, as defined previously (Yeo and Burge 2004; Krchnáková et al. 2019). The corresponding sequences were retrieved using BEDTools (Quinlan and Hall 2010) function “getfasta”. Sequence logos for 5′ and 3′ splice sites were generated using WebLogo (Crooks et al. 2004).

Furthermore, to verify the accuracy of our intron prediction, we used raw transcriptome and genome sequencing data, as well as the genome assembly and annotation of *P. papillatum* (Valach et al. 2023b). First, raw transcriptomic and genomic reads were analyzed using our pipeline to identify introns in *P. papillatum*, as described above for other diplonemids. Next, sequence logos were created using WebLogo (Crooks et al. 2004), as described above for the splice sites obtained using our pipeline and those derived from the previously published genome annotation of *P. papillatum* (Valach et al. 2023b).

### De novo intron motif and branch point analysis

To identify conserved sequence motifs within introns, we used the MEME suite (Bailey and Elkan 1994) (https://meme-suite.org/meme/tools/meme). For each diplonemid species, MEME was run separately on intronic sequences using classic mode with default parameters, a minimum motif width of 6, a maximum width of 50, and up to three motifs per run.

To identify potential branch point sequences, we used the seqkit locate command (Shen et al. 2016) to scan introns for the canonical YTRAY motif. The search was restricted to regions located more than 5 and <50 nt upstream of the 3′ splice site, and sequence logos were created using WebLogo (Crooks et al. 2004).

### snRNA identification and secondary structure prediction

Rfam covariance models were downloaded from Rfam (https://rfam.xfam.org/). The cmpress (INFERNAL v.1.1.3) program was run on Rfam.cm to prepare an input for cmscan program. Small nuclear RNAs (snRNAs) were identified using Infernal's cmscan program (Nawrocki and Eddy 2013) with the parameters “rfam”, “cut_ga”, and “nohmmonly”. Secondary structure models for snRNAs were generated using the R2R program v.1.0.5 (Weinberg and Breaker 2011).

### Identification of core spliceosome proteins

We established a custom snRNP database by acquiring core spliceosome proteins (Will and Lührmann 2011) associated with U1, U2, U4, U5, and U6 snRNAs from the National Center for Biotechnology Information at https://www.ncbi.nlm.nih.gov/. To identify potential candidate proteins, we conducted BLASTx searches (Altschul et al. 1990) using the above-mentioned proteins as queries and de novo assembled transcriptomes from each species as a database with the following parameters: *e*-value of 1 *×* 10^−10^, subject_besthit, max_target_seqs 3, and qcov_hsp_perc 25. To ensure the robustness of our findings, we executed reciprocal BLASTp searches (Altschul et al. 1990) with the following criteria: *e*-value of 1 *×* 10^−10^, max_target_seqs 1, and qcov_hsp_perc 30. Finally, we generated multiple sequence alignments of the candidate spliceosomal proteins using ClustalX v.2.1 (Thompson et al. 2002). Moreover, we used BLASTx to search for non-snRNP U2AF components by querying the de novo assembled transcriptomes of the six diplonemid species against *U2AF1* and *U2AF2* protein sequences obtained from NCBI, using an *e*-value cutoff of 1 *×* 10^−5^. Similarly, we also queried Lsm2–8 proteins, the Sm core proteins (SmB/B′, D1, D2, D3, E, F, and G), Dbr1, 65K/USP39, TXNL4A, SNU66/SART1, and core NTC components (PRP19, Celf1/CDC5L, Syf1 [NTC90], Syf2 [NTC31], Isy [NTC30], PRP1 [PRPG1], and Spf27 [NTC25]).

### Calculation of base-pairing probability

To investigate the base-pairing propensity between the 5′ and 3′ ends of introns across various species, intron sequences were extracted using the agat_sp_extract_sequences program (https://agat.readthedocs.io/en/latest/tools/agat_sp_extract_sequences.html). We extracted 9 base pair substrings from both the 5′ and 3′ ends of each intron, concatenating them into subsequences of 18 base pairs. Subsequently, we used the RNAfold v.2.4.18 (Lorenz et al. 2011) to predict the secondary structure of these sequences. Base-pairing probability (BPP) was calculated using the R programming language (R Core Team 2021), where we divided the total number of base-pairing nucleotides by the total number of nucleotide bases for each species. The ggplot2 package was used to create boxplots. The secondary structure prediction of introns was performed using RNAstructure v.6.4 (Reuter and Mathews 2010). For species *S. specki*, *L. lanifica*, *D. japonicum*, and *R. humris*, noncanonical introns (1.56%–2.89%) were filtered out due to their extremely low representation to minimize noise and ensure reliable BPP estimates. Similarly, BPP values were calculated for the exonic sequences spanning 9 nt upstream of the 5′ ss and 9 nt downstream from the 3′ ss for each species.

### Phylogenetic tree construction

We used a computational pipeline to find the evolutionary relationships inherent within canonical and noncanonical splice sites (5′ss and 3′ss) of *A. motanka, N. karyoxenos* with other marine diplonemids (*S. specki*, *L. lanifica*, *D. japonicum*, *R. humris*, and *P. papillatum*). Noncanonical introns were excluded for species *S. specki*, *L. lanifica*, *D. japonicum*, and *R. humris*, as they were poorly represented, allowing the phylogenetic analysis to focus on robust evolutionary signals. Initially, sequences were extracted from positions −3 to +9 at the exon–intron junction (consisting of three exonic positions and nine intronic positions) to represent the 5′ss motif. Similarly, positions −20 to +3 were extracted from the intron–exon junction (comprising 20 intronic positions and three exonic positions) to represent the 3′ss motif (Yeo and Burge 2004; Krchnáková et al. 2019). The conserved dinucleotide intron termini were removed from both 5′ss and 3′ss to prevent hiding of signals from other splice site positions. Subsequently, Bioconductor package Biostrings v.2.58.0 (Pagès et al. 2024) was used for construction of position weight matrices (PWMs) from the extracted canonical and noncanonical sequences at both 5′ss and 3′ss. Next, a distance matrix was computed using the PWMs, capturing the evolutionary distances between sequences. Hierarchical clustering was then applied to the distance matrix using the hclust function from the ape package v.5.7.1. (Paradis and Schliep 2019). Finally, the hierarchical clustering result was converted into a phylogenetic tree using the “as.phylo” (Paradis and Schliep 2019) function, and the resulting tree was visualized using the “plot” function. Furthermore, we constructed the species tree using FastTree implemented in OrthoFinder v.2.5.4 (Emms and Kelly 2019). For that, protein sequences from *A. motanka*, *N. karyoxenos*, *S. specki*, *L. lanifica*, *D. japonicum*, and *R. humris* were obtained by predicting open reading frames (ORFs) from their assembled transcripts using OrfPredictor (Min et al. 2005). Protein sequences for *P. papillatum* originated from the recently published genome annotation (Valach et al. 2023b). The species tree was generated by integrating OrthoFinder gene trees with the STAG (Emms and Kelly 2018) and STRIDE (Emms and Kelly 2017) algorithms. Phylogenomic tree was visualized with iTOL v.5 (Letunic and Bork 2021). To assess intron conservation, we compared transcripts between *A. motanka* and *N. karyoxenos*, using the recently sequenced, high-quality annotation of *P. papillatum* as a reference. Several homologous transcripts were identified by BLAST (Altschul et al. 1990), and intron–exon structures were examined in IGV (https://igv.org/) to determine whether intron positions were conserved and whether canonical introns had been replaced by noncanonical introns (Robinson et al. 2011).

### RT-PCR validation of introns

The RNA pellet was resuspended in nuclease-free water, treated with Turbo DNase (Thermo Fisher Scientific), and precipitated, and 1 µg of the total RNA was then used for reverse transcription. Reverse transcription was performed using SuperScript III (Thermo Fisher Scientific) and random hexamers. The cDNA was diluted 1:10 and analyzed by PCR using T100 Thermal Cycler (Bio-Rad) and Taq DNA Polymerase (Thermo Fisher Scientific). The primer sequences are provided in Supplemental Table S3.

### Analysis of splicing using reporter minigene assays

Procyclic stage *T. brucei* (SmOxP927 cell line) (Poon et al. 2012) were grown at 28°C in the SDM-79 medium (Life Technologies) supplemented with 10% fetal calf serum (Brun and Schonenberger 1979). Fragments of three genes from *N. karyoxenos* and one gene from *P. papillatum* were selected for experimentation. The HindIII and XbaI restriction sites were added to the 5′ and 3′ ends, respectively, of the *N. karyoxenos* gene fragments, while the corresponding ends of the *P. papillatum* gene fragment were furnished with the HindIII and BamHI sites. Along with the restriction sites, these fragments were synthesized (Life Technologies), and subsequently cloned into the expression plasmid pDEX777 (Poon et al. 2012). Stable SmOxP927-derived cell lines were prepared as described previously (Wirtz et al. 1999) with the following modifications. For each cell line, 6 μg of NotI-linearized pDEX777 plasmid was added to 1 × 10^7^ log-phase *T. brucei* cells, washed and resuspended in 0.5 mL of the transfection buffer (66 mM Na_2_HPO_4_; 23 mM NaH_2_PO_4_; 5 mM KCl, 50 mM Hepes pH 7.3), and transfected by three pulses (voltage 1.7 kV; pulse length 100 μsec; interval 100 msec; unipolar polarity) using the BTX ECM 830 electroporator. After electroporation, cells were added to 9.5 mL of SDM-79 and incubated at 28°C. After 20 h, phleomycin was added to the final concentration of 5 μg/mL, eventually leading to the selection of stable transfectants. The expression of gene fragments was induced by a 24 h incubation with doxycycline at the final concentration of 1 µg/mL.

### Isolation of total RNA from *Trypanosoma brucei*

1 × 10^8^ cells were spun for 10 min at 1500*g* at 4°C, washed with 1 mL PBS, resuspended in 1 mL TRIzol buffer, and incubated for 5 min at room temperature. Following the addition of 200 μL chloroform, the solution was homogenized and spun for 15 min at 20,000*g* at 4°C. Upon the transfer of the upper phase to a new tube, 15 μL of 3 M sodium acetate, 30 μg of glycogen, and 500 μL of 100% isopropanol were added and incubated at −20°C for 18 h. Next, the samples were centrifuged at 20,000*g* for 20 min, and the pellets were twice washed with 500 μL of 70% isopropanol and spun at 20,000*g* for 5 min between the washes. Finally, the pellets were resuspended in 50 μL of RNase-free distilled water.

## DATA DEPOSITION

The raw reads analyzed during the current study are available at DDBJ/ENA/GenBank under the following accession numbers: SRX5434882 and SRX5472370 (*A. motanka*), SRX5472384 and SRX5472372 (*D. japonicum*), SRX5434883 and SRX5472373 (*L. lanifica*), SRX5434880 and SRX5472374 (*N. karyoxenos*), SRX5434881 and SRX5472371 (*R. humris*), and SRX5434879 and SRX5472375 (*S. specki*). Intron coordinates are provided at https://doi.org/10.6084/m9.figshare.29656262.v1.

## SUPPLEMENTAL MATERIAL

Supplemental material is available for this article.
